# Debridement, Antibiotics, and Implant Retention (DAIR) Protocol for the Management of Early Periprosthetic Joint Infections: An Eight-Year Single-Centre Experience

**DOI:** 10.3390/jcm15103865

**Published:** 2026-05-17

**Authors:** Aleksandra Grajek, Sławomir Chaberek, Dariusz Grzelecki

**Affiliations:** 1 Prof. Adam Gruca Orthopedic and Trauma Teaching Hospital, Konarskiego 13, 05-400 Otwock, Poland; olagrajek@tlen.pl (A.G.); schaber@spskgruca.pl (S.C.); 2 Department of Orthopedics and Reconstructive Surgery, Centre of Postgraduate Medical Education, Prof. Adam Gruca Orthopedic and Trauma Teaching Hospital, Konarskiego 13, 05-400 Otwock, Poland

**Keywords:** infection, arthroplasty, hip, knee, DAIR, PJI

## Abstract

**Background**: This study aims to assess how patient comorbidities and risk factors influence treatment outcomes of periprosthetic joint infection (PJI). The role of timing for DAIR intervention, administration of antibiotics, and the microbiological profile in relation to infection recurrence were investigated. **Methods**: This retrospective study included 58 patients, 26 after total hip arthroplasty (THA) and 32 after total knee arthroplasty (TKA), who underwent surgery for early PJI managed with the complete DAIR protocol at a single academic orthopedic center (Professor Adam Gruca Orthopedic and Trauma Teaching Hospital) between January 2014 and January 2021. A minimum follow-up period after DAIR was five years. **Results**: In the overall cohort, therapeutic success was achieved in 41 of 58 patients (71%). Treatment of early PJI after THA was successful in 21 of 26 patients (81%), while after TKA, 20 of 32 patients (63%) achieved a favorable outcome. An increase in the number of comorbidities associated with infection risk was correlated with a lower likelihood of successful treatment using the DAIR protocol. Our analysis also demonstrated that the timing from total joint arthroplasty (TJA) to surgical intervention, the administration of antimicrobial therapy, and positive culture results influenced the success rate. **Conclusions**: The effectiveness of the DAIR protocol in managing early PJI is influenced by multiple factors. This study suggests that crucial determinants include prompt and accurate diagnosis, identification of patient-specific risk factors, the causative pathogen and its antibiotic administration, as well as the timing of intervention.

## 1. Introduction

Early periprosthetic joint infection (PJI) may arise soon after elective total joint arthroplasty (TJA) or manifest months to years later as a result of hematogenous dissemination of microorganisms [1,2]. The overall incidence of PJI is reported to range from 1% to 2%, with early PJI rates reaching up to 2.88% [3]. Currently, there is a lack of robust evidence to establish the optimal timing for implementing the Debridement, Antibiotics, and Implant Retention (DAIR) protocol in the management of early PJI. Recent investigations suggest that the most favorable outcomes with the DAIR protocol are observed when the procedure is conducted within 4 to 12 weeks post-TJA [4,5]. The success of this strategy is highly contingent upon multiple factors, particularly the formation of biofilms on the implant surface following microcolony development. Biofilm formation is influenced by environmental conditions, nutrient availability, the specific characteristics of the infecting pathogen, and the physicochemical properties of the implant surface [6,7,8].

With the global increase in primary total hip arthroplasty (THA) and total knee arthroplasty (TKA) procedures, the incidence of PJI is projected to rise correspondingly [9]. In cases of early PJI, timely and appropriate management strategies are critical for achieving effective infection control, improving clinical outcomes, and minimizing treatment costs [10]. Reported success rates for the Debridement, Antibiotics, and Implant Retention (DAIR) protocol vary significantly, ranging from 26% to 100%. These variations depend on cohort characteristics, follow-up duration, the specific joint involved, antibiotic regimen, and the criteria used to define therapeutic success [11]. Consequently, additional research is necessary to evaluate these variables and optimize treatment outcomes for early PJI.

The primary objective of this study is to determine the success rate of the DAIR protocol in managing early periprosthetic joint infections following primary total hip and knee arthroplasty at a single academic orthopedic center. Furthermore, the study aims to assess the impact of patient comorbidities and risk factors on treatment outcomes, as well as to investigate the influence of timing for DAIR intervention, and the microbiological profile of infecting pathogens on infection recurrence. Furthermore, to the best of our knowledge, this is the first study to comprehensively evaluate how antibiotic selection and administration impact the success rate of the DAIR protocol.

## 2. Materials and Methods

This study received approval from the Centre of Postgraduate Medical Education in Warsaw (Poland) Bioethics Committee (reference number 417/2023). A retrospective analysis was conducted on 58 patients—26 following THA and 32 following TKA—who underwent surgical intervention for early PJI managed with the complete Debridement, Antibiotics, and Implant Retention (DAIR) protocol at a single academic orthopedic center between January 2014 and January 2021. The minimum follow-up period for all patients was five years after completion of the DAIR procedure. All patients underwent a standardized DAIR protocol, which consisted of extensive surgical debridement, pulse lavage irrigation using a 0.9% sodium chloride solution, irrigation with 0.3% povidone-iodine—diluted with saline, Braunol^®^ (B. Braun Melsungen AG, Melsungen, Germany) for three minutes, exchange of mobile implant components, and administration of antibiotic therapy. In patients who received antibiotics, therapy was continued intravenously during 7–10 days (hospital stay) and oral antibiotics were administered up to six weeks postoperatively (DAIR procedure). Empiric antibiotic selection was guided by the cumulative antibiogram (microbiological map) prepared by the Hospital Infection Prevention and Control Team. Upon receipt of culture results, the established treatment protocol involved either switching to a pathogen-specific antibiotic or continuing the initial empiric antibiotic regimen, depending on the sensitivity profile and clinical assessment.

Inclusion criteria comprised confirmed early PJI, defined as occurring within six weeks of primary TJA. Patients with hematogenous early PJI were excluded, as all cases represented early postoperative PJI. Additional exclusions included patients with early infections following revision TJA, resection prostheses after tumor resections, periprosthetic fractures, dislocations, single-stage revisions for early PJI, or incomplete clinical data. In this study, therapeutic success following the DAIR protocol was defined as the complete eradication of infection, preservation of the implant, and achievement of pain-free, satisfactory joint function as assessed by the patient. Conversely, therapeutic failure was defined as the recurrence of infection and the necessity to perform an additional revision procedure. Demographic variables (age, sex, Body Mass Index [BMI]) and clinical characteristics (operated joint, presenting symptoms, infection risk factors, interval from TJA to DAIR, identified pathogen type, and antibiotic regimen) were extracted from the electronic medical records and are presented in Table 1.

### Statistical Analysis

Data management was performed using Microsoft Excel 365 (Microsoft, WA, USA), and statistical analyses were carried out with Statistica 13.3 (Tibco, CA, USA). The normality of continuous variable distributions was assessed using the Shapiro–Wilk test. Dichotomous variables were analyzed with Fisher’s exact test. For continuous variables not exhibiting a normal distribution, results were presented as medians with interquartile ranges (IQR) and evaluated using the Mann–Whitney U test. Relationships between study groups were explored via contingency tables: two-way tables were employed for analyses involving two variables, while multi-way tables were used for more complex comparisons across multiple variables. Statistical significance tests were selected according to group size and expected values, including the maximum likelihood (ML) Chi-square test, Chi-square test with Yates’ correction, and Fisher’s exact test. Given the retrospective design of this study and the inclusion of consecutive patients in the analysis, a post hoc power analysis was conducted regarding the timing of the DAIR procedure. The analysis demonstrated that the study had approximately 90% power (α = 0.05) to detect the observed association between the timing of DAIR revision and therapeutic outcomes. This level of power corresponds to a large effect size, as indicated by Cohen’s w = 0.54. A *p*-value less than 0.05 was considered indicative of statistical significance.

## 3. Results

Of the 58 patients who underwent the DAIR procedure, 2 individuals (3.5%) did not participate in postoperative outpatient follow-up and were therefore excluded from the final analysis. Prior to DAIR intervention, the cohort’s median C-reactive protein (CRP) level was 25.1 mg/L (interquartile range [IQR]: 14–52.1), and the median erythrocyte sedimentation rate (ESR) was 30 mm/h (IQR: 23.5–56.3). Overall, therapeutic success was achieved in 41 of 58 patients (71%). Notably, among patients treated for early PJI following THA, 21 of 26 (81%) demonstrated successful outcomes, whereas therapeutic success was observed in 20 of 32 (63%) patients after TKA. Symptoms occurring after primary joint replacement suggestive of early PJI were presented in Table 2.

### 3.1. Risk Factors

**Figure 1:**
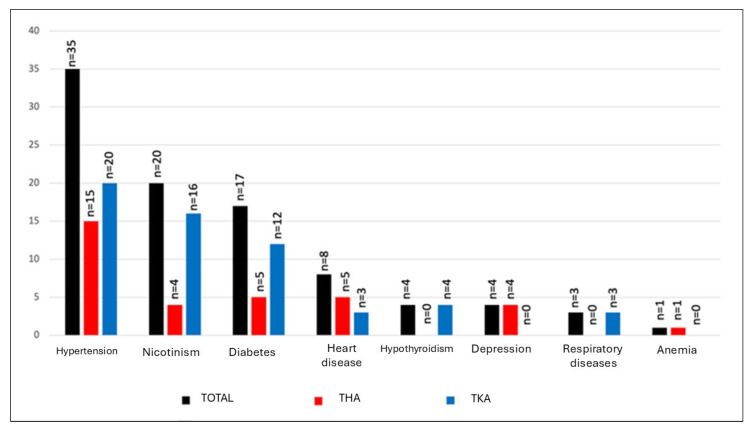
Risk factors of PJI. The total number of risk factors exceeds the number of patients due to the possibility of more than one risk factor of infection in a patient.

Therapeutic success was observed in all patients without comorbidities (n = 7, 100%). However, the probability of successful treatment decreased as the number of comorbidities increased: among patients with a single comorbidity, 17 of 19 (89.5%) achieved favorable outcomes; for those with two comorbidities, 13 of 18 (72.2%) were successfully treated; and only 4 of 10 patients (40%) with three comorbidities experienced therapeutic success. Importantly, no patients with four comorbidities attained a successful treatment outcome. Risk factors among all cohort were presented in Figure 1 and categorized by the number of comorbidities and success rate in each subgroup in Table 3.

### 3.2. Culturing

Preoperative joint aspiration and microbiological cultures were conducted in 32 patients. Positive culture results were obtained in 25 cases (78%), with pathogen concordance between preoperative and postoperative cultures observed in 20 patients. Among the sampling techniques utilized, sonication fluid demonstrated the highest postoperative culture accuracy at 77.2%, followed by tissue samples at 65.5%, and synovial fluid at 54.3% (see Table 4).

Statistical analyses demonstrated that there was no significant association between pathogen presence and the success rate of DAIR treatment (Chi-square test with Yates’ correction: *p* = 0.37; Pearson Chi-square test: *p* = 0.19; maximum likelihood Chi-square test: *p* = 0.21). Similarly, the virulence of pathogens in monomicrobial infections did not significantly influence treatment outcomes (Fisher’s exact test, two-tailed: *p* = 0.11; Pearson Chi-square test: *p* = 0.063; maximum likelihood Chi-square test: *p* = 0.059; Yates-corrected Chi-square test: *p* = 0.14). Coagulase-negative staphylococci (CNS) were the most frequently isolated pathogens overall (25.9%), particularly among cases following TKA (34.4%). In contrast, Staphylococcus aureus was most commonly identified in infections after THA. Polymicrobial infections, defined as the culture of two pathogens, were observed in 19% of cases, occurring more frequently in early knee PJIs (25%) compared to hip PJIs (11.6%). The overall false negative rate was 17.2%, with a higher frequency after THA (26.9%) than TKA (9.4%). Comprehensive microbiological culture results are provided in Table 5.

### 3.3. Timing

**Figure 2:**
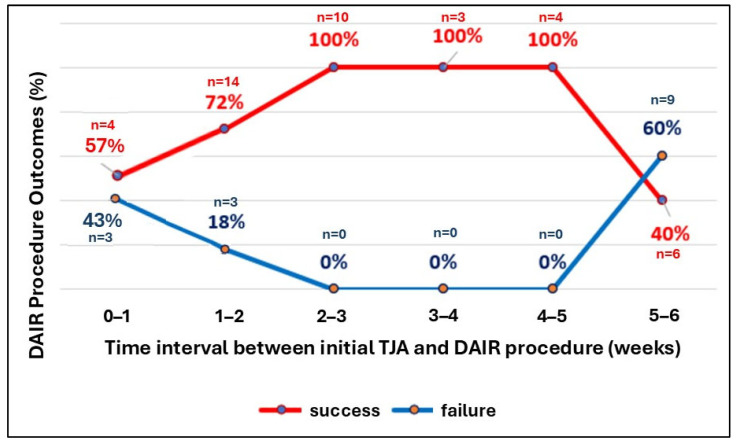
The effectiveness of the DAIR protocol depending on the time elapsed since the primary operation until revision surgery.

After excluding patients who did not attend postoperative follow-up appointments, therapeutic success following the DAIR protocol was achieved in 41 out of 56 patients (73.2%). This cohort included 21 of 25 individuals (84%) who underwent DAIR after THA and 20 of 31 patients (64.5%) following total knee arthroplasty (TKA). The distribution of successful and unsuccessful outcomes according to TJA type is depicted in Figure 2. Statistical analysis demonstrated a significant association between the timing of revision surgery and the probability of therapeutic success. Both the maximum likelihood Chi-square test (*p* = 0.003) and the Pearson Chi-square test (*p* = 0.01) confirmed the impact of the interval between the primary operation and the revision procedure on outcomes. Furthermore, rank correlation analysis (Kendall’s tau-c = 0.21) indicated a meaningful positive correlation between earlier revision and improved surgical results. Because of sparse cell counts in several subgroups, an exact Fisher’s test was subsequently conducted after categorizing the interval between primary arthroplasty and DAIR revision into two groups: ≤5 weeks and >5 weeks. The analysis showed that early revision was significantly associated with therapeutic success (35/38 vs. 6/15; Fisher’s exact test, *p* = 0.00017), with an odds ratio of 17.5 (95% CI: 3.65–83.91), strongly favoring earlier intervention.

### 3.4. Antimicrobial Treatment

**Figure 3:**
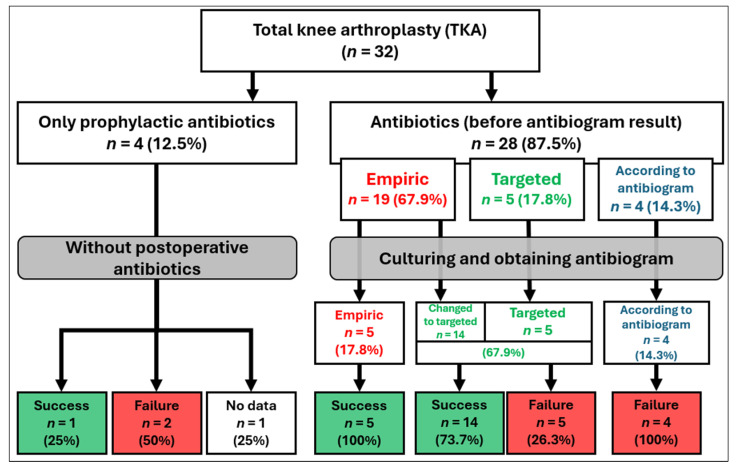
The impact of postoperative antibiotic therapy on the outcome of DAIR treatment after TKA.

**Figure 4:**
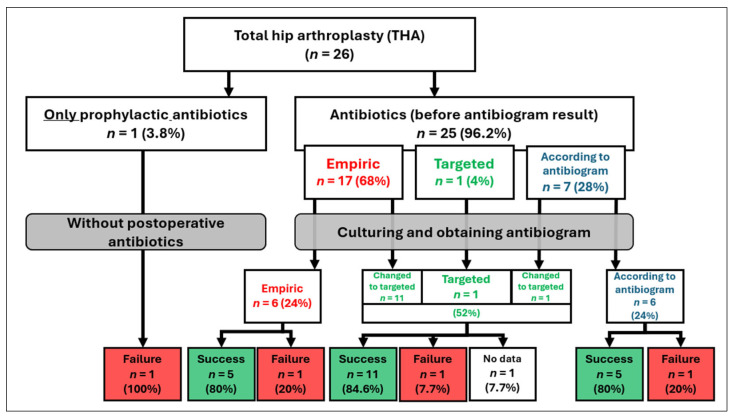
The impact of postoperative antibiotic therapy on the outcome of DAIR treatment after THA.

Post-revision, the vast majority of patients received antibiotic therapy, with 25 individuals (96.2%) following THA and 28 patients (87.5%) after TKA. Conversely, only one patient (3.8%) after THA and four patients (12.5%) following TKA were managed using perioperative prophylactic antibiotics alone. Among those administered targeted antibiotics, either prior to or subsequent to microbiological culture results, the therapeutic success rates were 84.6% for THA and 73.7% for TKA. Notably, patients treated exclusively with empiric antibiotics throughout the entire therapeutic period achieved success rates of 80% after THA and 100% following TKA. Treatment outcomes stratified according to antibiotic regimen are presented in Figure 3 and Figure 4.

**Figure 5:**
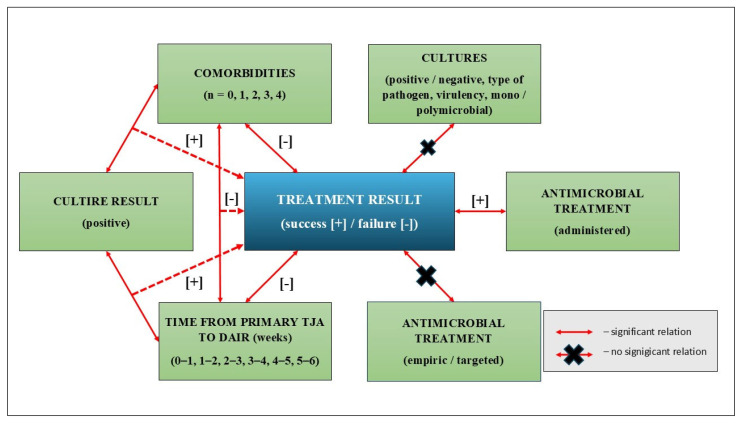
Significance of the relationships among the analyzed variables. [+] and [-] symbols represent positive and negative impact on the treatment result.

Statistical analysis revealed a significant association between the administration of antimicrobial therapy and the success of revision surgery, as evidenced by the Chi-square NW test (*p* = 0.045) and the Pearson Chi-square test (*p* = 0.039). Additional factors contributing to the outcomes of DAIR treatment are depicted in Figure 5.

## 4. Discussion

Periprosthetic joint infections pose a significant threat to joint function, often resulting in substantial deterioration of patient quality of life. Management of PJI typically requires multiple surgical interventions and prolonged hospitalization, imposing a considerable financial burden on healthcare systems [12,13]. Despite increasing research interest in PJI, the majority of studies concentrate on chronic infections, whereas investigations addressing early-stage infections remain scarce [14,15,16,17]. Prompt intervention and implementation of the debridement, antibiotics, and implant retention (DAIR) protocol have demonstrated improved therapeutic outcomes. Nevertheless, reported DAIR success rates for early PJI are highly variable, and consensus regarding the optimal treatment approach is lacking. In the present study, the elevated failure rate of DAIR observed in PJIs following TKA compared to THA may be attributable to the knee joint’s more complex biomechanics, a greater tendency for persistent biofilm formation, increased prevalence of highly virulent pathogens, and a higher risk of chronic infection among TKA patients, as previously described by Longo et al. [10]. In a comprehensive meta-analysis encompassing 81 studies, Abbaszadeh et al. reported an overall success rate of 64.1% for the DAIR protocol in the management of PJI [15]. Specifically, the success rate for early PJI following primary joint replacement was 65.8%, whereas early hematogenous PJIs demonstrated a slightly lower success rate of 60.9%. In contrast, the efficacy of DAIR for late PJIs declined markedly to 26.4%. The analysis further indicated that outcomes for early PJI were superior following THA compared to TKA, corroborating findings from the present study [18]. In a retrospective study conducted by Chandler et al., in a retrospective investigation of patients with acute hematogenous PJIs treated at a single institution, utilized Kaplan–Meier survival analysis and found that 75% of patients remained infection-free five years after DAIR intervention. Notably, 70.7% of treatment failures occurred within the first postoperative year, underscoring the critical importance of vigilant early postoperative monitoring and timely intervention to optimize long-term outcomes [19]. Rahardja et al., in a prospective multicenter cohort study involving 189 DAIR-treated patients, observed a therapeutic success rate of only 45% following TKA, with outcomes closely associated with the timing of surgical intervention. The ICM 2025 recommendations advocate for DAIR to be performed within 6 weeks of the primary TJA and within 7 days of infection symptom onset [20]. Löwik et al. further stratified success rates according to timing, reporting the highest success rate (71%) when DAIR was undertaken between the fifth and sixth weeks after infection onset, while effectiveness during other intervals reached up to 58% [21]. These results are consistent with institutional data from our center, which demonstrated optimal outcomes when the DAIR procedure was performed between the second and fifth weeks following primary surgery. Of note, the curve depicting treatment effectiveness exhibited a parabolic trend, emphasizing the significance of intervention timing. Importantly, even when DAIR was performed outside the conventional 6-week window—specifically between the seventh and twelfth weeks postoperatively—a relatively high success rate of 58% was observed. This suggests that, in selected cases, effective infection management with implant retention may be achievable beyond the standard early PJI timeframe. Therefore, individualized patient assessment is warranted, and the DAIR approach may remain a viable option for up to three months after symptom onset, contingent upon patient-specific factors and clinical judgment.

In the present analysis, no statistically significant association was observed between the presence of individual comorbidities and the therapeutic success of PJI management using the DAIR protocol. Kuiper et al., in a cohort study of 91 patients treated with DAIR for PJI, reported that single systemic conditions—including diabetes mellitus, cardiovascular disease, and chronic obstructive pulmonary disease—did not significantly affect treatment outcomes in univariate analysis [22]. Nonetheless, the authors proposed that these comorbidities may indirectly contribute to treatment failure by impairing immune function and delaying tissue repair. Their multivariate analysis identified infection onset more than two years post-implantation, elevated erythrocyte sedimentation rate (ESR > 60 mm/h), and central nervous system (CNS) infection as independent risk factors for DAIR failure. Similarly, Lora-Tamayo et al., in a multicenter cohort study comprising 345 PJI cases managed with DAIR, found that comorbidities such as diabetes mellitus, chronic kidney disease, and ischemic heart disease were not independent predictors of treatment failure [23]. However, patients with these conditions exhibited increased rates of relapse and prolonged wound healing. In contrast, Tsang et al., in a prospective observational study, demonstrated that DAIR treatment was significantly less effective in patients with internal comorbidities compared to those without chronic illnesses [24].

This study demonstrates that implant sonication yields the highest rate of positive cultures in early PJI, with 77.2% of cases showing pathogen detection. This finding aligns with Trampuz et al., who reported that sonication significantly enhances pathogen identification compared to conventional tissue or synovial fluid cultures [25]. Piper et al. further highlighted that positive cultures obtained after implant sonication are particularly valuable for identifying biofilm-producing organisms, such as *Staphylococcus epidermidis*, which may be missed by standard synovial fluid cultures. In this analysis, tissue cultures from the prosthesis area were positive in 61.5% of patients [26]. These results are consistent with recent studies by Watanabe et al. and Wouthuyzen-Bakker et al., both of whom emphasize the critical role of tissue cultures in diagnosing PJI, especially for infections caused by low-virulence pathogens [27,28]. The rate of positive preoperative synovial fluid cultures was 78.2%, notably higher than intraoperative fluid cultures (54.3%), with a 60% concordance between these results. This underscores the importance of synovial fluid analysis in the early diagnosis of PJI. Li et al. found that preoperative synovial fluid and intraoperative culture sensitivities matched in 81.29% of cases [29]. In contrast, van Schaik et al., in a systematic review of seven studies involving 1677 patients, observed that concordance rates for synovial fluid cultures obtained preoperatively and intraoperatively varied from 52% to 79% [30]. Variability in findings across studies may be attributed to differences in sample collection methods, transportation conditions, culture techniques, infection characteristics (monomicrobial versus polymicrobial), and pathogen type. These factors highlight the importance of standardized protocols and careful interpretation of culture results to optimize diagnostic accuracy for PJI.

Precise identification of the causative pathogen is crucial for tailoring antibiotic therapy and optimizing the efficacy of the DAIR protocol. In cases where cultures yield negative results, clinicians are compelled to employ empirical antibiotic regimens, which may adversely affect treatment outcomes. Multiple studies have demonstrated that culture-negative infections are correlated with a higher risk of DAIR protocol failure. In the current study, negative culture results were observed in 17.2% of cases, consistent with previously reported rates ranging from 5% to 40% [31,32]. Importantly, administration of antibiotics prior to microbiological sample collection can significantly increase the probability of false-negative culture results [33]. The latest 2025 ICM guidelines recommend that antibiotics should generally be administered in the operating room immediately before surgical intervention and sample collection, except when urgent therapy is clinically warranted [34]. Additionally, prompt initiation of empirical antibiotic therapy following revision surgery directly influences the likelihood of successful treatment outcomes [35].

A notable limitation of this study is the relatively small patient cohort. Although the analysis includes individuals who underwent surgery at a single institution over an eight-year period. Given the limited sample size, the potential for type II errors, and reduced statistical power, the results of this study should be interpreted with caution. This is especially important when considering findings related to specific antibiotic subgroups and the influence of comorbidity burden. Moreover, early PJIs are rare complications that are often challenging to diagnose in the immediate postoperative phase, as their clinical manifestations may be obscured by postoperative inflammatory responses. Consequently, certain cases may initially go undetected and are subsequently managed as delayed PJIs, utilizing alternative treatment strategies.

## 5. Conclusions

The efficacy of the DAIR protocol in the management of early PJI is contingent upon several critical determinants. Accurate and prompt diagnosis of infection, comprehensive evaluation of patient-specific risk factors, definitive identification of the causative organism and its antimicrobial susceptibility profile, optimal timing for the initiation of antibiotic therapy, and thorough surgical debridement are all indispensable for achieving favorable clinical outcomes. In light of the multifaceted nature and variability of these factors, there is a compelling need for additional research, specifically prospective, multicenter investigations with larger patient populations, to more precisely delineate evidence-based practices and enhance therapeutic success.

## Figures and Tables

**Table 1:** Demographic and clinical data summarized using medians (IQR) for non-normally distributed values.

	TOTAL (*n* = 58)	THA (*n* = 26)	TKA (*n* = 32)	*p*-Value
**Sex (Female/Male)**	34/24	12/14	22/10	0.11 **
**Age (years)**	69 (63.3–73)	69 (63.3–73)	69.5 (63.3–73)	0.61 *
**Body mass (kg)**	83 (76.3–93.5)	84.5 (80–93.3)	80 (75–92.8)	0.3 *
**BMI (kg/m^2^)**	26.3 (25.7–27.1)	25.9 (25.4–26.3)	27 (26–27.5)	0.35 *
**CRP before TJA (mg/L)**	2.6 (1.3–5.2)	2.8 (1.7–5.1)	2.1 (1.1–5.2)	0.45 **
**ESR before TJA (mm/h)**	10 (5–21.5)	10 (5–15)	10 (6–25)	0.45 **
**Surgery time (primary) [min]**	85 (80–104)	76 (66.3–88.8)	95 (78.8–106.3)	0.03 **
**Time to discharge from TJA (days)**	7 (6–7.8)	6 (5–7.8)	7 (6–7.3)	0.22 **
**CRP before DAIR (mg/L)**	25.1 (14–52.1)	20.5 (8.7–37.4)	25.6 (19–62)	0.95 *
**ESR before DAIR (mm/h)**	30 (23.5–56.3)	42.5 (23.5–67.5)	30 (22.5–50)	0.83 *

* Student *T*-test; ** Mann–Whitney U test.

**Table 2:** Symptoms occurring after primary joint replacement suggestive of early PJI. The total number of symptoms exceeds the number of patients due to the possibility of more than one symptom of infection in a patient.

Preoperative PJI Symptoms	Patients (*n* = 58)	(%)
**Wound leakage**	24	41
**Increased joint temperature**	3	5
**Skin redness**	17	29
**Wound dehiscence**	6	10
**Significant joint pain**	19	33
**Body temperature > 38 °C**	8	14
**Joint swelling**	2	3
**Fistula**	2	3
**Fluid collection (confirmed by ultrasound)**	3	5

**Table 3:** Data showcasing patients categorized by the number of comorbidities (risk factors), the corresponding percentage of comorbidities, and the therapeutic success rate of the DAIR protocol within each subgroup.

No. of Comorbidities	No. of Patients	% of All Patients	Therapeutic Success of DAIR (%)
0	7	12.5	100
1	19	33.9	89.5
2	18	32.1	72.2
3	2	3.6	40
4 and more	10	17.9	0

**Table 4:** Results of microbiological cultures depending on the type of the testing material.

	Total	THA	TKA
**Sonication Fluid**	44 positive to 57 cultured (77.2%)	17 positive to 26 cultured (65.4%)	27 positive to 31 cultured (87.1%)
**Tissues**	38 positive to 58 cultured (65.5%)	16 positive to 26 cultured (61.5%)	22 positive to 32 cultured (68.75%)
**Synovial F** **l** **uid**	25 positive to 46 cultured (54.3%)	8 positive to 20 cultured (40%)	17 positive to 26 cultured (65.4%)
**Total**	107 positive to 161 cultured (66%)	41 positive to 72 cultured (57%)	66 positive to 89 cultured (74%)

**Table 5:** Microbiological culture results.

Pathogen	Total (*n* = 58)	THA (*n* = 26)	TKA (*n* = 32)
***Staphylococcus* CN**	**15 (25.9%)**	**4 (15.4%)**	**11 (34.4%)**
MRCNS	11 (73.3%)	3 (75%)	8 (72.7%)
*St. epidermidis*	9	*2*	*7*
*St. haemolyticus*	2	1	*1*
MSCNS	4 (26.7%)	1 (25%)	3 (27.3%)
*St. epidermidis*	3	*1*	*2*
*St. cohnii*	1	*0*	*1*
***Staphylococcus aureus***	**9 (15.5%)**	**5 (19.2%)**	**4 (12.5%)**
MSSA	6 (66.7%)	4 (80%)	2 (50%)
MRSA	3 (33.3%)	1 (20%)	2 (50%)
**Gram (−) pathogens**	**5 (8.6%)**	**3 (11.6%)**	**2 (6.25%)**
*Proteus mirabilis*		*0*	*1*
*Enterobacter* spp.		*1*	*1*
*Klebsiella pneumoniae*		*2*	*0*
***Enterococcus faecalis***	**3 (5.2%)**	**1 (3.8%)**	**2 (6.25%)**
***Streptococcus* spp.**	**3 (5.2%)**	**1 (3.8%)**	**2 (6.25%)**
*Streptococcus gr* “*viridans*”		*0*	*2*
*Streptococcus gr* “*G*”		*1*	*0*
***Corynebacterium* spp.**	**1 (1.7%)**	**1 (3.8%)**	**0 (0%)**
***Cutibacterium acnes***	**1 (1.7%)**	**1 (3.8%)**	**0 (0%)**
**2 pathogens**	**11 (19%)**	**3 (11.6%)**	**8 (25%)**
*MSSA/MRCNS*		*0*	*1*
*MSSA, MSCNS*		*1*	*0*
*MSSA/Streptococcs gr* “*viridans*”		*0*	*1*
*MSSA/Enterococcus faecalis*		*1*	*3*
*MRCNS/Enterococcus faecalis*		*0*	*2*
*P. aeruginosa*/*P. mirabilis*		*0*	*1*
*Enterobacter cloacae/Enterococcus faecalis*		*1*	*0*
**False negative**	**10 (17.2%)**	**7 (26.9%)**	**3 (9.4%)**

## Data Availability

The data presented in this study are available on request from the corresponding author.

## Abbreviations

The following abbreviations are used in this manuscript:

BMI	Body Mass Index
DAIR	Debridement, Antibiotics and Implant Retention
PJI	Periprosthetic Joint Infection
THA	Total Hip Arthroplasty
TKA	Total Knee Arthroplasty
TJA	Total Joint Arthroplasty
